# Prognostic value of pretreatment neutrophil-to-lymphocyte ratio in breast cancer patients receiving neoadjuvant chemotherapy: a systematic review and meta-analysis

**DOI:** 10.3389/fonc.2026.1849765

**Published:** 2026-05-29

**Authors:** Ziqian Zhao, Haoyi Xu, Hongyi Yuan, Binlin Ma, Chao Dong

**Affiliations:** The Clinical Medical Research Center of Breast and Thyroid Tumor in Xinjiang, Tumor Hospital Affiliated to Xinjiang Medical University, Urumqi, China

**Keywords:** breast cancer, meta-analysis, neoadjuvant chemotherapy, neutrophil-to-lymphocyte ratio, prognosis

## Abstract

**Objective:**

This study examined whether pretreatment neutrophil-to-lymphocyte ratio (NLR) was associated with survival outcomes and treatment response in breast cancer patients treated with neoadjuvant chemotherapy.

**Methods:**

PubMed, EMBASE, Web of Science, and the Cochrane Library were searched for eligible studies published up to April 2026. Using Stata 18.0 and Review Manager 5.4, pooled hazard ratios (HRs) and odds ratios (ORs) with 95% confidence intervals (CIs) were synthesized to determine the relationship of pretreatment NLR with overall survival (OS), disease-free survival (DFS), recurrence-free survival (RFS), and pathologic complete response (pCR).

**Results:**

Fifty-six studies involving 16,539 patients were included. Elevated pretreatment NLR was significantly associated with worse OS (HR = 1.89, 95% CI 1.48–2.41; P<0.00001), DFS (HR = 1.79, 95% CI 1.45–2.23; P<0.00001), and RFS (HR = 1.89, 95% CI 1.17–3.05; P = 0.01), as well as a reduced likelihood of achieving pCR (OR = 0.50, 95% CI 0.40–0.64; P<0.00001). These findings remained stable in sensitivity analyses restricted to multivariable-adjusted estimates.

**Conclusion:**

Elevated pretreatment NLR was associated with poorer survival outcomes and lower pCR rates after NACT. However, because of substantial heterogeneity and inconsistent cutoffs, NLR should be viewed as a complementary marker requiring prospective validation rather than a routine prognostic factor.

## 1 Introduction

Breast cancer (BC) continues to be the most frequently diagnosed cancer in women across the world, with approximately 2.308 million new cases recorded in 2022 (1). For individuals with locally advanced disease, NACT has become an established treatment strategy because tumor burden can be reduced and the feasibility of breast-conserving surgery can be increased (2). Nevertheless, treatment response varies substantially across individuals. Although pCR is generally regarded as being associated with favorable long-term outcomes, and patients who achieve pCR have been reported to have an approximately 30% higher 5-year DFS rate than those with residual disease after treatment (3), only 19%–30% of patients ultimately reach this endpoint. In addition, conventional clinicopathologic parameters remain limited in their ability to predict treatment benefit before therapy. Therefore, readily available and noninvasive markers that may help predict survival and treatment response before therapy are still needed.

One potential approach is to assess systemic inflammatory status using routine blood-based indicators. Among these, NLR is a widely studied and easily obtainable marker of systemic inflammation and host immune status (4). Established tumor- and immune-related predictors, including molecular subtype, PD-L1 expression, and tumor-infiltrating lymphocytes (TILs), provide important information on tumor biology and the local immune microenvironment. Unlike these tissue-based or subtype-specific markers, NLR can be measured from routine peripheral blood before treatment. Thus, NLR may provide complementary information when evaluating prognosis and treatment response in patients receiving NACT. Several studies have explored its prognostic relevance in individuals with BC receiving NACT (5). In a single-center cohort of 1,097 patients, Bae et al. reported that elevated pretreatment NLR predicted worse OS, DFS, and a lower pCR rate after 4 years of follow-up (6). In another multicenter retrospective study, Azab et al. analyzed 123 patients with triple-negative breast cancer treated with NACT between 2007 and 2014 and reported that a high pretreatment NLR was linked to inferior OS (7). Despite these observations, the clinical value of NLR in the neoadjuvant setting has not yet been clearly established.

A meta-analysis published by Zhou et al. in 2021 comprised 19 cohorts published between 2014 and 2020 and showed that raised NLR was linked to shorter OS, DFS, and a lower pCR rate (8). Since then, a substantial body of new clinical studies have been published, and their findings have not been entirely consistent. Moreover, studies reporting RFS were not included in that earlier analysis. These limitations may have affected the generalizability and robustness of the available evidence. This updated meta-analysis added 37 newly published studies and further explored the relationship of pretreatment NLR with survival outcomes, including RFS, in breast cancer patients receiving NACT.

## 2 Materials and methods

### 2.1 Literature search

The present meta-analysis followed the Preferred Reporting Items for Systematic Reviews and Meta-Analyses (PRISMA) statement (9). The literature was searched in PubMed, Embase, Web of Science, and the Cochrane Library, and the final search update was completed on April 1, 2026. No language restriction was applied. The search included terms related to neutrophils, lymphocytes, breast cancer, and neoadjuvant chemotherapy. The full PubMed search strategy is listed in **Supplementary Table 1**.

### 2.2 Study selection

Study screening was based on the PICOS framework, including population, intervention, comparison, outcomes, and study design. Studies were included if patients had pathologically confirmed breast cancer, received NACT or chemotherapy-based neoadjuvant treatment, were stratified according to pretreatment NLR level, and reported associations of NLR with OS, DFS, RFS, or pCR using HRs or ORs with 95% CIs, or provided sufficient data for calculation. For studies reporting pCR, total pCR or overall pCR, defined as the absence of residual invasive disease in both the breast and axillary lymph nodes (ypT0/is ypN0 or equivalent), was extracted preferentially when available. Studies were excluded when they involved hematologic malignancies, were reviews or other non-original publications, failed to report HRs for OS, DFS, or RFS or ORs for pCR, or assessed NLR only as a continuous variable. For studies from the same center with overlapping populations, only the largest dataset was retained. Two authors independently screened the titles, abstracts, and full texts, and consensus was reached through discussion when disagreements arose.

### 2.3 Data extraction

A predefined Excel spreadsheet was used for data collection. Two reviewers extracted the relevantinformation independently, and the extracted data were then checked by a third reviewer.Disagreements were resolved through discussion. The collected information included first author, publication year, country, study center, study design, sample size, patient age, study population, study period, treatment method, follow-up duration, TNM stage, NLR cutoff value, timing of NLR measurement, definition of pCR in the original study, and HRs or ORs with 95% CIs for OS, DFS, RFS, and pCR. The extracted pCR definitions were summarized in **Supplementary Table 7**. When the original study did not clearly define pCR, the definition was recorded as “not clearly reported” rather than inferred by the reviewers. When both univariable and multivariable estimates were available, the multivariable-adjusted results were used preferentially.

### 2.4 Quality assessment

The quality of the eligible studies was evaluated with the Newcastle–Ottawa Scale (NOS),which covers selection, comparability, and outcome. All studies scored more than 5 points,suggesting acceptable quality (**Supplementary Table 2**). Two reviewers independently performed the assessment, and any differences were settled through discussion.

### 2.5 Statistical analysis

HRs for OS and DFS, and ORs for pCR, together with 95% CIs, were obtained from each included study. Pooled effect estimates were obtained using fixed-or random-effects framework depending on heterogeneity across reports. Heterogeneity was quantified with Cochran’s Q test and the Higgins I² statistic (10). Substantial heterogeneity was defined as I²>50% or P<0.05. A fixed effects approach was chosen when heterogeneity was not notable; otherwise, a random-effects approach was adopted. Potential sources of heterogeneity were explored using subgroup analyses and meta-regression according to available study-level variables, including sample size, region, age, study population, tumor stage, follow-up duration, NLR cutoff value, and adjustment method. Robustness was examined using leave-one-out sensitivity analyses. Publication bias was inspected by funnel plots and Egger’s test; when significant asymmetry was observed, trim-and-fill analyses were conducted to obtain bias-adjusted effects. All tests were two-sided, and P<0.05 was defined significant. Statistical analyses were completed with using STATA (version 18.0) and Review Manager (version 5.4).

## 3 Results

### 3.1 Study selection and characteristics

**Figure 1:**
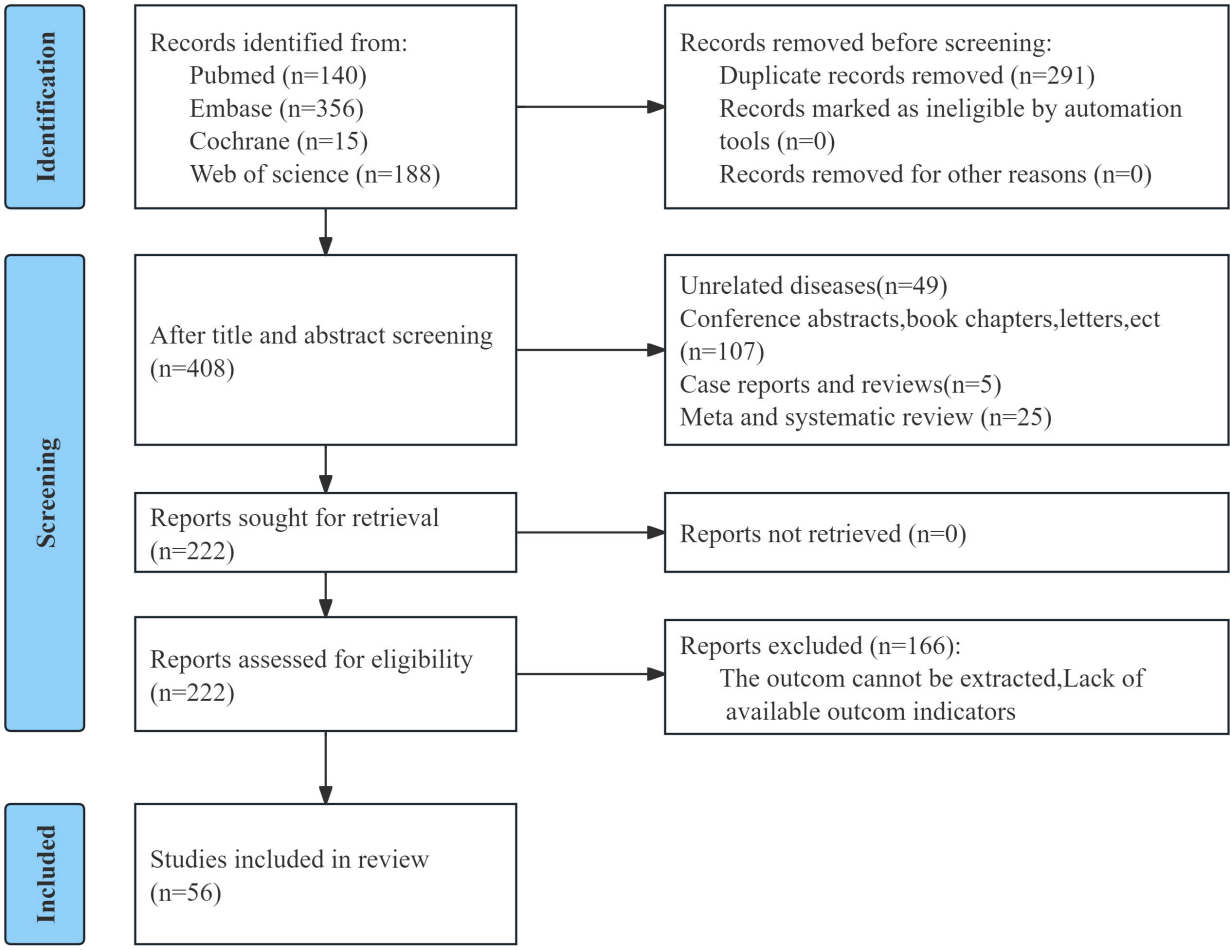
PRISMA flow diagram of study selection. The flow diagram shows the process of literature identification, duplicate removal, title and abstract screening, full-text assessment, and final inclusion. A total of 56 studies involving 16,539 patients were included in the final meta-analysis.

The database search initially screened 699 records. After duplicates were removed, 408 records remained for title and abstract screening, during which 186 records were omitted. After full-text assessment of 222 articles, 166 were not included in the final analysis. As a result, 56 studies involving 16,539 patients were retained in the final meta-analysis (**Figure 1**) (5–7, 11–63). Sample sizes ranged from 46 to 1,097.

The 56 studies were released between 2014 and 2026, 35 were undertaken in Asia, 11 were conductedin non-Asian regions, and the remaining 10 were multicenter cohorts. All patients receivedneoadjuvant chemotherapy (NACT), and NLR was measured before treatment initiation. All included cohorts were published in English and had a retrospective design, with study periods spanning from 1996 to 2024. The median age varied from 36 to 65 years, and the NLR cutoff values spanned 0.13 to 4.09. Nineteen studies reported data on OS, 20 on DFS, 6 on RFS, and 31 on pCR. In view of the relatively large proportion of cohorts from China and Turkey, an additional cross-checking table was constructed to examine the risk of cohort overlap on the basis of the first author, study center or hospital, study period, patient population or subtype, and outcome overlap risk (**Supplementary Table 3**). Most studies reported multivariable-adjusted estimates for NLR. In contrast, univariable estimates were used in 5 studies for OS (13, 16, 22, 36, 43), 8 studies for DFS (13, 16, 17, 32, 37, 41, 43, 57), 3 studies for RFS (20, 22, 36), and 9 studies for pCR (11, 21, 31, 38, 45, 46, 48, 55, 57) (**Table 1**).

**Table 1:** Baseline characteristics of the included studies evaluating pretreatment NLR in breast cancer patients receiving NACT.

Author	Study period	Region	Study design	Population	Treatment method	Timing of detection	No. of patients	Median follow-up (months)	Mean/median Age	TNM stage	Cut-off	Outcomes
Acikgoz, O. 2022 (5)	2014-2019	Turkey	Retrospective cohort	Locally advanced breast cancer (LABC)	NACT	Pretreatment	139	39.5	45.0	II-III	2.4	pCR
Alan, O. 2020 (11)	2015-2017	Turkey	Retrospective cohort	Locally advanced and metastatic breast cancer	NACT	Pretreatment	55	41.0	48.5	I-IV	3.3	pCR
Alshamsan, B. 2024 (12)	2005-2014	Egypt	Retrospective cohort	Locally advanced breast cancer (LABC)	NACT and surgery	Pretreatment	465	77.0	44.0	I-III	2.2	OS/DFS/pCR
Arici, M.O. 2024 (13)	2015-2023	Turkey	Retrospective cohort	Early or locally advanced breast cancer	NACT and surgery	Pretreatment	304	38.5	50.0	II-III	2.1	OS/DFS/pCR
Azab, B. 2021 (7)	2007-2014	Multicenter	Retrospective cohort	TNBC	NACT and surgery	Pretreatment	123	54.0	52.9	I-IV	2.0	OS/DFS
Bae, S.J. 2020 (6)	2007-2018	Korea	Retrospective cohort	Non-metastatic, HER2-negative breast cancer	NACT and surgery	Pretreatment	1097	56.0	47.0	I-III	2.7	OS/DFS/pCR
Baskurt, K. 2026 (14)	2022-2024	Turkey	Retrospective cohort	HR +/HER2 − BC	NACT	Pretreatment	96	16.6	65.0	I-IV	1.7	pCR
Chae, S. 2018 (15)	2004-2012	Korea	Retrospective cohort	TNBC	NACT and surgery	Pretreatment	87	57.0	45.8	I-IV	1.7	pCR
Chen, L. 2020 (16)	1999-2014	China	Retrospective cohort	Advanced BC	NACT and surgery	Pretreatment	262	43.4	48.0	II-III	2.5	OS/DFS
Chen, X.W. 2025 (17)	2019-2023	China	Retrospective cohort	TNBC	NACT and surgery	Pretreatment	112	13.0	47.5	II-IIIC	2.7	DFS/pCR
Chen, Y. 2016 (18)	2001-2010	China	Retrospective cohort	Primary breast cancer	NACT and surgery	Pretreatment	347	55.0	46.4	I-IV	2.1	RFS
Cherifi, F. 2022 (19)	2014-2020	Multicenter	Retrospective cohort	HER2-low early BC	NACT and surgery	Pretreatment	1047	30.0	51.0	I-IIIB	2.0	OS/RFS
Choi, H. 2020 (20)	2000-2014	Korea	Retrospective cohort	Locally advanced breast cancer	NACT and surgery	Pretreatment	148	70.0	47.3	I-III	0.1	RFS
Chung, W.S. 2022 (21)	2012-2019	China	Retrospective cohort	Primary non-metastatic TNBC	NACT and surgery	Pretreatment	88	NA	50.8	T1-T3	1.5	pCR
Corbeau, I. 2020 (22)	2009-2023	Multicenter	Retrospective cohort	Early Breast Cancer	NACT	Pretreatment	280	80.4	50.3	I-II	2.0	OS/RFS
Dan, J.Q. 2020 (23)	2012-2017	China	Retrospective cohort	Primary breast cancer	NACT and surgery	Pretreatment	242	NA	50.0	II-III	3.1	pCR
Dong, J. 2021 (24)	2015-2020	China	Retrospective cohort	Primary breast cancer	NACT and surgery	Pretreatment	241	NA	48.0	NA	1.8	pCR
Dong, X. 2021 (25)	2010-2014	China	Retrospective cohort	TNBC	NACT and surgery	Pretreatment	170	34.0	40.0	NA	1.9	DFS
Ebaid, N.F. 2025 (26)	2019-2024	Egypt	Retrospective cohort	BC	NACT and surgery	Pretreatment	46	NA	45.5	NA	1.8	pCR
Eren, T. 2020 (27)	2009-2018	Turkey	Retrospective cohort	Locally advanced breast cancer (LABC)	NACT and surgery	Pretreatment	131	NA	49.0	I-III	2.0	pCR
Gao, S. 2023 (28)	2009-2018	China	Retrospective cohort	BC	NACT and surgery	Pretreatment	421	43.2	49.0	II-III	2.2	OS
García, M.E.G. 2026 (29)	2009-2019	Multicenter	Retrospective cohort	Early-stage breast cancer	NACT and surgery	Pretreatment	801	NA	49.0	I-III	1.9	pCR
Geng, S.K. 2018 (30)	2002-2014	China	Retrospective cohort	BC	NACT and surgery	Pretreatment	96	24.0	53.8	I-III	1.9	DFS
Gong, Y.C. 2025 (31)	1999-2018	China	Retrospective cohort	BC	NACT and surgery	Pretreatment	244	NA	48.9	NA	2.6	pCR
Goto, W. 2018 (32)	2007-2015	Japan	Retrospective cohort	BC	NACT and surgery	Pretreatment	239	40.8	56.0	IIA/IIB/IIIA	1.6	DFS
Grassadonia, A. 2021 (33)	2004-2019	Multicenter	Retrospective cohort	Luminal breast cancer	NACT and surgery	Pretreatment	168	95.8	50.0	I-III	2.1	OS/DFS
Guo, Q. 2025 (34)	2000-2018	China	Retrospective cohort	TNBC	NACT and surgery	Pretreatment	422	80.0	50.1	II-III	1.7	OS
Huang, W.L. 2023 (35)	2014-2018	China	Retrospective cohort	BC	NACT and surgery	Pretreatment	260	40.0	46.5	I-III	4.0	DFS
Hutajulu, S.H. 2025 (36)	2018-2022	Indonesia	Retrospective cohort	BC	NACT	Pretreatment	202	46.0	51.0	I-III	2.0	OS/RFS
Jiang, C, X. 2022 (37)	2012-2016	China	Retrospective cohort	BC	NACT and surgery	Pretreatment	305	NA	49.0	I-III	2.2	OS/DFS
Karaali, C. 2025 (38)	2010-2021	Turkey	Retrospective cohort	BC	NACT	Pretreatment	228	NA	50.0	I-III	2.2	pCR
Koh, Y.W. 2014 (39)	2002-2010	South Korea	Retrospective cohort	HR +/HER2 − BC	NACT and surgery	Pretreatment	157	21.0	44.0	I-III	2.3	OS/RFS
Kusama, H. 2023 (40)	2013-2019	Japan	Retrospective cohort	TNBC	NACT and surgery	Pretreatment	266	NA	52.5	I-III	2.6	pCR
Lee, J. 2019 (41)	2007-2015	Korea	Retrospective cohort	TNBC	NACT	Pretreatment	50	37.9	50.0	I-III	3.2	DFS
Li, F.C. 2024 (42)	2011-2023	China	Retrospective cohort	Young patients with breast cancer	NACT and surgery	Pretreatment	215	75.8	36.0	I-III	1.6	pCR
Li, X.M. 2021 (43)	2008-2018	China	Retrospective cohort	Primary breast cancer	NACT and surgery	Pretreatment	282	63.0	50.0	I-III	1.8	OS/DFS
Lokesh, K.N. 2026 (44)	2022-2024	India	Retrospective cohort	TNBC	NACT	Pretreatment	101	NA	49.0	II-III	2.2	pCR
Lou, C.Y. 2022 (45)	2015-2021	China	Retrospective cohort	TNBC	NACT	Pretreatment	92	NA	52.3	II-III	3.1	pCR
Ma, R. 2023 (46)	2019-2022	China	Retrospective cohort	BC	NACT and surgery	Pretreatment	112	NA	50.9	I-IV	2.0	pCR
Ma, Y.Z. 2021 (47)	2017-2018	China	Retrospective cohort	BC	NACT	Pretreatment	203	31.0	46.6	II-III	3.0	DFS
Pang, J. 2021 (48)	2010-2018	China	Retrospective cohort	Locally advanced TNBC	NACT	Pretreatment	310	NA	NA	I-III	1.9	pCR
Polho, G.B. 2025 (49)	2012-2024	Multicenter	Retrospective cohort	TNBC	NACT	Pretreatment	692	59.6	48.5	II-III	2.0	OS/pCR
Rubovszky, G. 2026 (50)	2010-2018	Hungary	Retrospective cohort	TNBC	NACT	Pretreatment	137	86.2	52.0	II-III	2.8	OS/DFS
Sahin, A.B. 2021 (51)	2008-2019	Turkey	Retrospective cohort	BC	NACT	Pretreatment	743	67.5	48.0	T1-T4	2.3	pCR
Song, D.B. 2022 (52)	2016-2018	China	Retrospective cohort	BC	NACT and surgery	Pretreatment	144	32.0	50.4	I-III	2.4	DFS
Sun, Y. 2025 (53)	2010-2020	China	Retrospective cohort	Invasive breast cancer	NACT	Pretreatment	209	NA	50.9	I-IV	1.5	pCR
Tang, L. 2022 (54)	2012-2019	China	Retrospective cohort	HR +/HER2 − BC	NACT	Pretreatment	273	NA	49.8	I-III	2.5	pCR
Van Berckelaer, C. 2021 (55)	1996-2016	Multicenter	Retrospective cohort	Inflammatory breast cancer (IBC)	NACT	Pretreatment	125	NA	56.6	III	2.6	pCR
Wang, C. 2024 (56)	2014-2019	China	Retrospective cohort	TNBC	NACT	Pretreatment	83	69.0	46.5	I-III	2.7	OS
Wu, X.L. 2026 (57)	2015-2023	Multicenter	Retrospective cohort	HER2-positive breast cancer	NACT and surgery	Pretreatment	224	47.1	47.6	I-III	1.7	DFS/pCR
Yang, S.H. 2024 (58)	2011-2017	Multicenter	Retrospective cohort	BC	NACT	Pretreatment	323	NA	52.3	I-III	4.1	OS/DFS
Yao, L. 2023 (59)	2013-2020	Multicenter	Retrospective cohort	Invasive breast cancer	NACT and surgery	Pretreatment	784	NA	50.7	I-III	1.8	pCR
Yildirim, S. 2024 (60)	2010-2022	Turkey	Retrospective cohort	BC	NACT	Pretreatment	624	42.0	50.0	I-III	2.3	pCR
Yoon, T.I. 2026 (61)	2007-2013	South Korea	Retrospective cohort	BC	NACT	Pretreatment	1052	112.0	45.5	I-III	2.3	DFS
Zhao, M. 2023(62)	2017-2018	China	Retrospective cohort	TNBC	NACT and surgery	Pretreatment	126	37.0	50.1	I-IV	3.0	OS
Zhu, J.J. 2021 (63)	2014-2019	China	Retrospective cohort	Invasive breast cancer	NACT and surgery	Pretreatment	346	NA	48.0	II-III	1.7	pCR

BC, breast cancer; TNBC, triple-negative breast cancer; HR, hormone receptor; HER2, human epidermal growth factor receptor 2; LABC, locally advanced breast cancer; IBC, inflammatory breast cancer; NACT, neoadjuvant chemotherapy; NLR, neutrophil-to-lymphocyte ratio; OS, overall survival; DFS, disease-free survival; RFS, recurrence-free survival; pCR, pathological complete response; NA, not available or not reported.

### 3.2 Study quality

NOS scores were at least 5 in all included studies, indicating overall acceptable methodological quality (**Supplementary Table 1**).

### 3.3 Meta-analysis results

The detailed subgroup analysis results for OS, DFS, RFS, and pCR are summarized in **Table 2**.

**Table 2:** Subgroup analyses of the associations between elevated pretreatment NLR and clinical outcomes after NACT.

Subgroup	OS				DFS				RFS				pCR			
	Study group	HR [95%CI]	*P* value	*I* ^2^	Study group	HR [95%CI]	*P* value	*I* ^2^	Study group	HR [95%CI]	*P* value	*I* ^2^	Study group	OR [95%CI]	*P* value	*I* ^2^
Total	19	1.89 [1.48, 2.41]	<0.00001	56%	20	1.79 [1.45, 2.23]	<0.00001	60%	6	1.89 [1.17, 3.05]	0.01	71%	31	0.50 [0.40, 0.64]	<0.00001	83%
Sample size																
<300	10	2.46 [1.32, 4.57]	0.005	76%	14	1.92 [1.31, 2.82]	0.0008	63%	4	2.28 [0.95, 5.50]	0.07	83%	21	0.39 [0.26, 0.59]	<0.00001	86%
≥300	9	1.66 [1.40, 1.97]	<0.00001	0%	6	1.61 [1.31, 1.98]	<0.00001	49%	2	1.63 [1.16, 2.29]	0.005	0%	10	0.67 [0.53, 0.84]	0.0005	66%
Follow-up																
<50 months	7	2.25 [1.31, 3.84]	0.003	61%	11	2.01 [1.41, 2.88]	0.0001	40%	3	1.88 [0.99, 3.58]	0.05	61%	7	0.40 [0.25, 0.62]	<0.0001	60%
≥50 months	10	1.79 [1.27, 2.52]	0.0009	63%	7	1.70 [1.18, 2.46]	0.004	78%	3	2.03 [0.88, 4.73]	0.1	83%	6	0.56 [0.43, 0.75]	<0.0001	32%
Mean/median age																
<50	8	1.77 [1.34, 2.33]	<0.0001	36%	10	1.76 [1.37, 2.26]	<0.00001	59%	2	5.66 [2.25, 14.27]	0.0002	37%	18	0.42 [0.31, 0.58]	<0.00001	80%
≥50	11	1.97 [1.31, 2.96]	0.001	66%	10	1.76 [1.16, 2.68]	0.008	63%	4	1.35 [1.05, 1.74]	0.02	0%	12	0.60 [0.38, 0.94]	0.03	87%
Region																
Asia(0)	10	2.10 [1.45, 3.03]	<0.0001	51%	13	1.67 [1.31, 2.15]	<0.0001	53%	4	2.47 [1.18, 5.19]	0.02	77%	16	0.42 [0.27, 0.65]	0.0001	84%
Non-Asia(1)	3	2.00 [1.36, 2.92]	0.0004	6%	3	1.83 [1.24, 2.71]	0.003	37%	NA	NA	NA	NA	10	0.49 [0.31, 0.77]	0.002	75%
Multicenter(2)	6	1.57 [0.96, 2.55]	0.07	72%	4	1.83 [0.71, 4.75]	0.21	81%	2	1.30 [0.75, 2.24]	0.35	48%	5	0.74 [0.56, 0.98]	0.04	76%
NLR cut-off																
<2	4	1.62 [1.19, 2.22]	0.002	0%	5	2.63 [1.36, 5.11]	0.004	70%	3	2.38 [0.88, 6.43]	0.09	79%	13	0.41 [0.28, 0.62]	<0.0001	84%
≥2	15	2.00 [1.46, 2.74]	<0.0001	64%	15	1.66 [1.33, 2.08]	<0.00001	56%	3	1.67 [0.92, 3.04]	0.09	72%	18	0.56 [0.40, 0.78]	0.0007	82%
Population																
All	5	1.83 [1.38, 2.43]	<0.0001	0%	9	1.43 [1.22, 1.67]	<0.00001	0%	2	1.43 [1.02, 2.01]	0.04	0%	11	0.51 [0.34, 0.75]	0.0008	67%
TNBC	6	2.92 [1.55, 5.51]	0.001	75%	5	2.98 [1.47, 6.02]	0.002	64%	NA	NA	NA	NA	8	0.46 [0.23, 0.90]	0.02	89%
Other specific breast cancer	4	1.78 [0.64, 4.91]	0.27	77%	3	1.42 [0.48, 4.21]	0.53	81%	2	2.49 [1.18, 5.26]	0.02	50%	6	0.48 [0.29, 0.80]	0.005	81%
Stage-specific breast cancer	4	1.35 [0.98, 1.86]	0.07	3%	3	1.47 [1.12, 1.93]	0.005	0%	2	3.00 [0.32, 28.42]	0.34	91%	6	0.51 [0.28, 0.90]	0.02	87%
Tumor stage																
Non-metastatic	17	1.70 [1.39, 2.10]	<0.00001	39%	18	1.62 [1.32, 1.94]	<0.00001	42%	5	2.09 [1.08, 4.04]	0.03	77%	23	0.61 [0.48, 0.77]	<0.0001	82%
Mixed	2	10.80 [1.85, 62.97]	0.008	59%	1	5.21 [2.35,11.55]	<0.0001	NA	1	1.57 [1.05, 2.35]	0.03	NA	5	0.24 [0.16, 0.37]	<0.00001	6%
Adjustment																
Univariate analysis	5	1.40 [0.99, 1.98]	0.06	16%	8	1.49 [1.17, 1.91]	0.001	11%	3	1.92 [0.70, 5.25]	0.2	83%	9	0.63 [0.37, 1.07]	0.09	88%
Multivariate analysis	14	2.10 [1.55, 2.83]	<0.00001	61%	12	1.99 [1.46, 2.71]	<0.0001	71%	3	1.97 [1.25, 3.12]	0.004	43%	22	0.45 [0.35, 0.59]	<0.00001	78%

HRs were used for OS, DFS, and RFS, whereas ORs were used for pCR. For survival outcomes, an HR >1 indicates poorer survival in patients with elevated pretreatment NLR. For pCR, an OR <1 indicates a lower likelihood of achieving pCR in patients with elevated pretreatment NLR. “No. of cohorts” refers to the number of cohorts included in each subgroup analysis. NA indicates that data were not available or that the subgroup analysis was not applicable. Other specific breast cancer populations:non-metastatic, HER2-negative breast cancer;HR+/HER2− BC;HER2-low early BC;luminal breast cancer;young patients with breast cancer;inflammatory breast cancer (IBC);HER2-positive breast cancer. Stage-specific breast cancer populations:locally advanced breast cancer (LABC);locally advanced breast cancer;early or locally advanced breast cancer;Early Breast Cancer;early-stage breast cancer;advanced BC;locally advanced and metastatic breast cancer.

#### 3.3.1 NLR and OS

**Figure 2:**
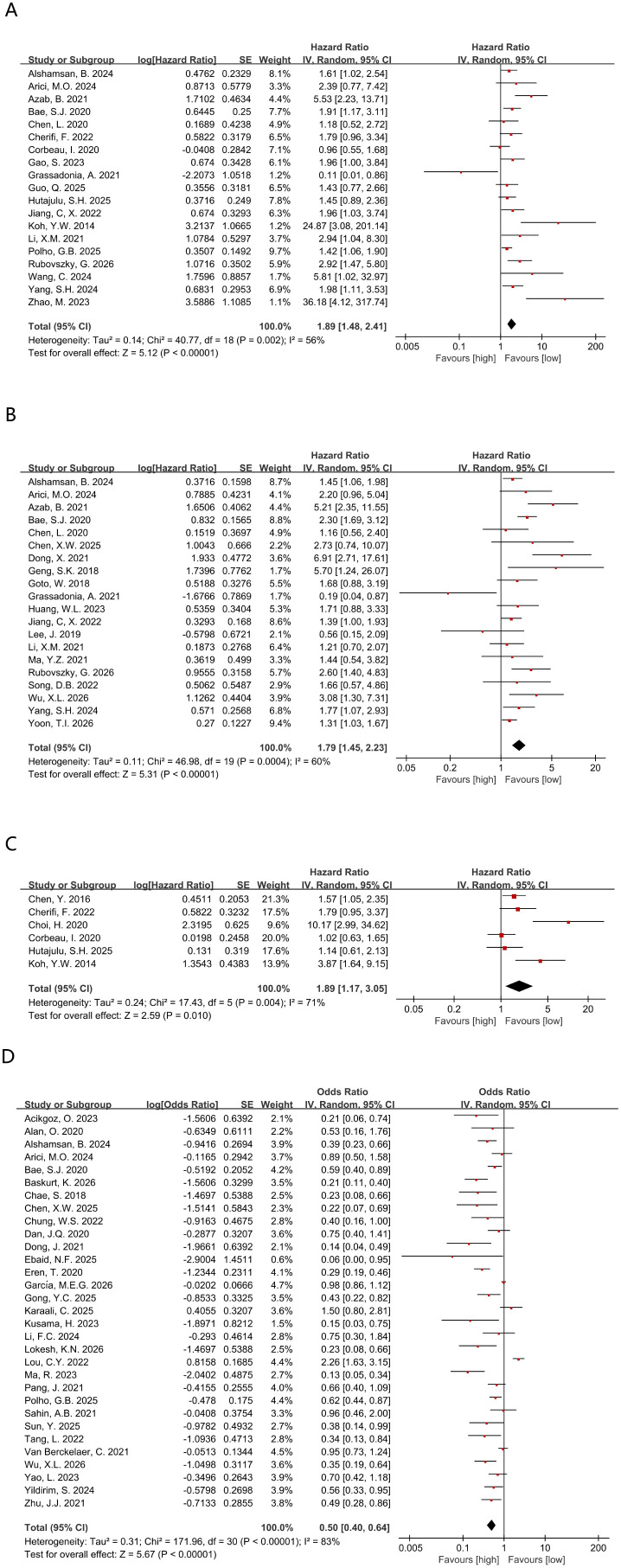
Forest plots of the associations between elevated pretreatment NLR and clinical outcomes after NACT. Panels **(A–D)** show OS, DFS, RFS, and pCR, respectively. HRs were used for survival outcomes, and ORs were used for pCR. HR >1 indicates poorer survival, whereas OR <1 indicates a lower likelihood of achieving pCR in patients with elevated pretreatment NLR. NLR, neutrophil-to-lymphocyte ratio; NACT, neoadjuvant chemotherapy; OS, overall survival; DFS, disease-free survival; RFS, recurrence-free survival; pCR, pathological complete response; HR, hazard ratio; OR, odds ratio.

Nineteen cohorts were included in the OS analysis. Because substantial heterogeneity was observed (I²=83%), a random-effects model was used. Elevated pretreatment NLR was associated with worse OS (HR = 1.89, 95% CI 1.48–2.41; P<0.00001; **Figure 2A**). Subgroup analyses showed generally consistent directions of association, although heterogeneity remained in several strata. Tumor stage was identified as a potential source of heterogeneity; the clinically relevant summary is presented in **Table 3**, and detailed meta-regression results are provided in **Supplementary Table 4**.

**Table 3:** Summary of potential sources of heterogeneity identified in subgroup and meta-regression analyses.

Variables	Coefficient	P value	95% CI	R²	τ²
Sample size					
Per 1-patient increase	-0.00030	0.351	[-0.00094,0.00034]	-60.20%	0.052
Follow-up					
Per 1-month increase	-0.013	0.082	[-0.028,0.002]	-343.20%	0.144
Mean/Median age					
Per 1-year increase	0.019	0.626	[-0.058,0.097]	-20.10%	0.039
NLR cut-off					
Per 1-unit increase	0.207	0.126	[-0.058,0.473]	97.96%	0.00066
Region					
Non-Asia vs Asia	0.061	0.819	[-0.459,0.580]	-17.30%	0.038
Multicenter vs Asia	-0.214	0.296	[-0.614,0.187]		
Population					
Triple-negative breast cancer vs Unselected breast cancer	0.201	0.476	[-0.352,0.754]	-161.70%	0.085
Other specific breast cancer vs Unselected breast cancer	-0.040	0.906	[-0.694,0.615]		
Stage-specific breast cancer vs Unselected breast cancer	-0.335	0.267	[-0.925,0.256]		
Tumor stage					
Mixed vs Non-metastatic	1.498	0.001	[0.647,2.349]	100%	0
Adjustment					
Multivariate vs Univariate	0.315	0.124	[-0.086,0.716]	19.14%	0.026

This table presents univariable meta-regression results for OS. The coefficient represents the change in the log HR associated with each covariate. For categorical variables, the first category listed in the comparison was used as the reference group. R² indicates the proportion of between-study heterogeneity explained by the covariate, and τ² represents the residual between-study variance. Results should be interpreted cautiously because meta-regression was based on study-level data.

#### 3.3.2 NLR and DFS

Twenty cohorts were included in the DFS analysis. A random-effects model was used because moderate to substantial heterogeneity was present (I²=60%). Elevated pretreatment NLR was associated with poorer DFS (HR = 1.79, 95% CI 1.45–2.23; P<0.00001; **Figure 2B**). Most subgroup estimates showed a similar direction of association, but heterogeneity was not fully resolved. Study population and tumor stage may partly explain between-study variability; the clinically relevant summary is presented in **Table 3**, and detailed meta-regression results are provided in **Supplementary Table 5**.

#### 3.3.3 NLR and RFS

Six cohorts were included in the RFS analysis. Elevated pretreatment NLR was associated with poorer RFS (HR = 1.89, 95% CI 1.17–3.05; P = 0.01; **Figure 2C**). Heterogeneity was also present in this analysis (I²=71%). Because only six cohorts were available, subgroup estimates were unstable and meta-regression was not performed. Therefore, the RFS result should be considered less stable than the OS, DFS, and pCR findings, as summarized in **Table 3**.

#### 3.3.4 NLR and pCR

Thirty-one cohorts were included in the pCR analysis. Because substantial heterogeneity was observed (I²=83%), a random-effects model was used. Elevated pretreatment NLR was associated with a lower likelihood of achieving pCR (OR = 0.50, 95% CI 0.40–0.64; P<0.00001; **Figure 2D**). Most subgroup estimates were directionally consistent with the main result, although heterogeneity remained. Tumor stage and NLR cutoff value may have contributed to this variability; the clinically relevant summary is presented in **Table 3**, and detailed meta-regression results are provided in **Supplementary Table 6**.

### 3.4 Sensitivity analysis

Leave-one-out sensitivity analyses were performed as supplementary robustness checks. The pooledestimates for OS, DFS, RFS, and pCR were not materially changed after sequential exclusion ofindividual studies, suggesting that no single study substantially drove the overall findings (**Supplementary Figure 3**). Sensitivity analyses restricted to multivariable-adjusted estimates also showed results consistent with the main analyses (**Supplementary Figure 1**).

### 3.5 Publication bias

Publication bias was assessed using funnel plots together with Egger’s and Begg’stests (**Supplementary Figure 4**). No obvious publication bias was detected for DFS or RFS, whereas possible publication biaswas observed for OS and pCR. Trim-and-fill analyses did not materially change the pooled estimatesfor OS or pCR (**Supplementary Figure 2**), suggesting that the main findings were not driven solely by potential publication bias.

## 4 Discussion

The central message of this meta-analysis is that pretreatment NLR was associated with poorer survival outcomes and lower pCR rates in breast cancer patients receiving NACT, but this association should be interpreted cautiously. The included studies showed substantial heterogeneity, used different NLR cutoffs, and were mostly retrospective. Therefore, NLR should be viewed as a simple and low-cost complementary marker of prognosis and treatment response, rather than as a stand-alone or ready-to-use mainstream prognostic factor.

Compared with the meta-analysis published by Zhou et al. (8), the present analysis offered a broader and more up-to-date synthesis of the available evidence. Zhou et al. found that elevated NLR was related to poorer OS, DFS, and pCR in individuals with breast cancer undergoing neoadjuvant treatment. In the current analysis, the prognostic association was extended to RFS, suggesting that the clinical value of NLR may be wider than previously recognized. This discrepancy may be related to several differences in study design and evidence selection.Most importantly, the current analysis included studies published through April 2026, which expanded the overall sample size and increased the diversity of the study populations. More multicenter cohorts and more data from non-Asian populations were incorporated, which may strengthen the stability and generalizability of the pooled estimates. In addition, stricter eligibility criteria were used to improve consistency in biomarker definition and to reduce the risk of overlapping populations. For instance, the earlier meta-analysis included a study evaluating derived NLR (dNLR) reported by Li et al. (64). Because dNLR is defined differently from conventional NLR, combining the two measures in the same pooled analysis may not be methodologically appropriate.Another important difference is that the literature search conducted by Zhou et al. ended in 2021, and later studies were therefore not represented. In the present study, possible overlap between the 2022 report by Jiang, C.X. et al. (37) and the 2020 report by Jiang, C. et al. (65) was further examined because both appeared to originate from the same center during similar study periods. To avoid duplicate inclusion as much as possible, the more recent report was retained. Furthermore, more extensive subgroup analyses were carried out according to region, sample size, NLR cutoff value, follow-up duration, mean age, adjustment status, study population, and tumor stage across the outcomes of OS, DFS, RFS, and pCR. Univariable and multivariable meta-regression analyses were also performed for OS, DFS, and pCR to further investigate potential sources of heterogeneity. Taken together, these methodological differences may partly explain why the evidence of the present study were not fully compatible with those of the earlier meta-analysis.

As summarized in **Table 3**, subgroup and meta-regression analyses suggested that heterogeneity was partly related to tumor stage, study population, and NLR cutoff value. Tumor stage appeared to influence the OS analysis, whereas study population and tumor stage may have contributed to heterogeneity in DFS. For pCR, tumor stage and NLR cutoff value were potential sources of heterogeneity. Because only six cohorts reported RFS, meta-regression was not performed for this outcome. These findings indicate that the association between NLR and outcomes may vary according to disease extent, patient composition, and the cutoff used to define high NLR. Therefore, NLR should be interpreted as a complementary marker rather than as a marker with a uniform effect across all patient groups.

Although elevated NLR was connected with unfavorable survival endpoints in this study, its clinical utility is more likely to lie in its role as an integrated indicator of host systemic status rather than as a direct mechanistic driver of tumor progression. NLR is generally regarded as a marker of systemic inflammatory and immune status. Both processes are closely linked to tumor progression, treatment tolerance, and survival. Its association with prognosis may therefore reflect the biological state of the host rather than a direct causal role of NLR itself. Lymphocytes contribute substantially to antitumor immunity by recognizing (66) and limiting tumor growth and dissemination (67). By contrast, neutrophils can promote tumor progression and metastasis through the release of inflammatory mediators, including matrix metalloproteinase-9 (MMP-9), neutrophil elastase (NE), and interleukin-8 (IL-8) (68). From this perspective, NLR is best interpreted as a composite reflection of the host’s overall systemic condition rather than as a direct surrogate for any single biological pathway. Therefore, NLR should not be interpreted as a replacement for established predictors such as molecular subtype, PD-L1 expression, or TILs. Instead, it may serve as a peripheral blood-based indicator that complements tumor-based biomarkers, particularly when tissue-based immune assessment is unavailable or incomplete.

Several limitations should also be acknowledged. First, substantial heterogeneity was observedacross studies, which may affect the robustness and generalizability of the pooled estimates.Although subgroup analyses, meta-regression, sensitivity analyses, and prediction intervals were used to explore this issue, residual confounding and unexplained between-study variability cannot be fully excluded. Therefore, the pooled estimates should be interpreted as evidence of association rather than as direct evidence for clinical implementation. Notably, the 95% prediction intervals for DFS, RFS, and pCR crossed the null value, suggesting that the strength of the observed associations may vary considerably across future clinical settings. In addition, because only a few studies reported RFS, the ability to identify stable effect modifiers for this outcome remained limited. Although OS was commonly reported and therefore retained in the analysis, it is not breast cancer-specific; more specific endpoints, such as breast cancer-specific survival, invasive disease-free survival, or breast cancer-free survival, were inconsistently reported and could not be pooled. Second, most included studies were retrospective, and the possibility of selection bias and measurement bias therefore remains. Furthermore, important clinical variables, including details of neoadjuvant treatment, were often insufficiently reported, which limits deeper interpretation of the results. In addition, treatment evolution, subtype heterogeneity, and differences in pCR reporting should be considered when interpreting the results. Detailed NACT regimens were not consistently available, preventing reliable treatment-era or regimen-specific analyses. Although pCR definitions were checked and summarized in **Supplementary Table 7**, residual heterogeneity related to pCR definition and limited subtype-specific data could not be fully excluded. Third, NLR may be influenced by infection, comorbid conditions, medication use, and interlaboratory differences in testing methods, but these factors were often not adequately documented or adjusted for in the original studies. Inconsistent reporting of the exact NLR calculation method, measurement units, and conversion approaches may also have contributed to between-study heterogeneity. Post-treatment NLR and longitudinal changes in NLR were not synthesized because only limited studies reported these metrics, and the definitions and sampling time points varied substantially. Fourth, the NLR cutoff values used across the included studies varied substantially, ranging from 0.1258 to 4.09, and a universally accepted threshold is still lacking. This variability reduces comparability between studies and may hinder the broader clinical use of NLR in different patient populations. As a result, current evidence remains insufficient to justify the routine adoption of a single cutoff value in clinical practice. Fifth, although subgroup analyses were conducted by breast cancer subtype or study population, data for several individual molecular subtypes were limited, which weakened the strength of subtype-specific conclusions. Finally, although the trim-and-fill analysis for pCR produced an adjusted estimate similar to the original result, Egger’s test still indicated possible publication bias or small-study effects. These findings suggest that the observed association should be interpreted cautiously, as the effect size may have been overestimated by unpublished studies with negative results.

Future large-scale, multicenter prospective studies are still required to harmonize NLR calculation and cutoff definitions and to collect detailed data on treatment regimens, complications, and concomitant medications in a more systematic manner. This will help clarify the prognostic role of NLR in individuals with BC receiving NACT. Further work should also examine whether interventions targeting inflammation, nutritional status, or immune function can improve patients’ overall systemic condition, and whether longitudinal changes in NLR provide prognostic information beyond a single pretreatment assessment.

## 5 Conclusion

Overall, elevated pretreatment NLR was connected with lower pCR and poorer survival outcomes in individuals with BC receiving NACT. These findings indicate that NLR may serve as a simple complementary prognostic indicator, although standardized cutoff values and prospective validation are still required before routine clinical application.

## Data Availability

The original contributions presented in the study are included in the article/**Supplementary Material**. Further inquiries can be directed to the corresponding authors.

## Supplementary material

Supplementary Figure 1 Forest plots of multivariable-adjusted associations between elevated pretreatment NLR and clinical outcomes after NACT. Panels A–D show OS, DFS, RFS, and pCR, respectively. HRs were used for survival outcomes, whereas ORs were used for pCR.

Supplementary Figure 2 Trim-and-fill analyses assessing the potential impact of publication bias. Panel A shows the analysis for OS, and panel B shows the analysis for pCR.

Supplementary Figure 3 Leave-one-out sensitivity analyses of the associations between elevated pretreatment NLR and clinical outcomes after NACT. Panels A–D show OS, DFS, RFS, and pCR, respectively.

Supplementary Figure 4 Funnel plots for publication bias in the analyses of elevated pretreatment NLR and clinical outcomes after NACT. Panels A–D show OS, DFS, RFS, and pCR, respectively.

Supplementary Table 1 Full PubMed search strategy.

Supplementary Table 2 Quality assessment of the included studies using the Newcastle–Ottawa Scale.

Supplementary Table 3 Cross-checking table for potential cohort overlap among included studies.

Supplementary Table 4 Univariable and multivariable meta-regression analyses for OS.

Supplementary Table 5 Univariable and multivariable meta-regression analyses for DFS.

Supplementary Table 6 Univariable and multivariable meta-regression analyses for pCR.

Supplementary Table 7 Definitions of pathological complete response reported in the included studies.
