# Supplementary figures and images for: Prognostic value of pretreatment neutrophil-to-lymphocyte ratio in breast cancer patients receiving neoadjuvant chemotherapy: a systematic review and meta-analysis
Source: Front Oncol. 2026 May 29;16:1849765. doi: 10.3389/fonc.2026.1849765 (PMC13260012; doi:10.3389/fonc.2026.1849765)

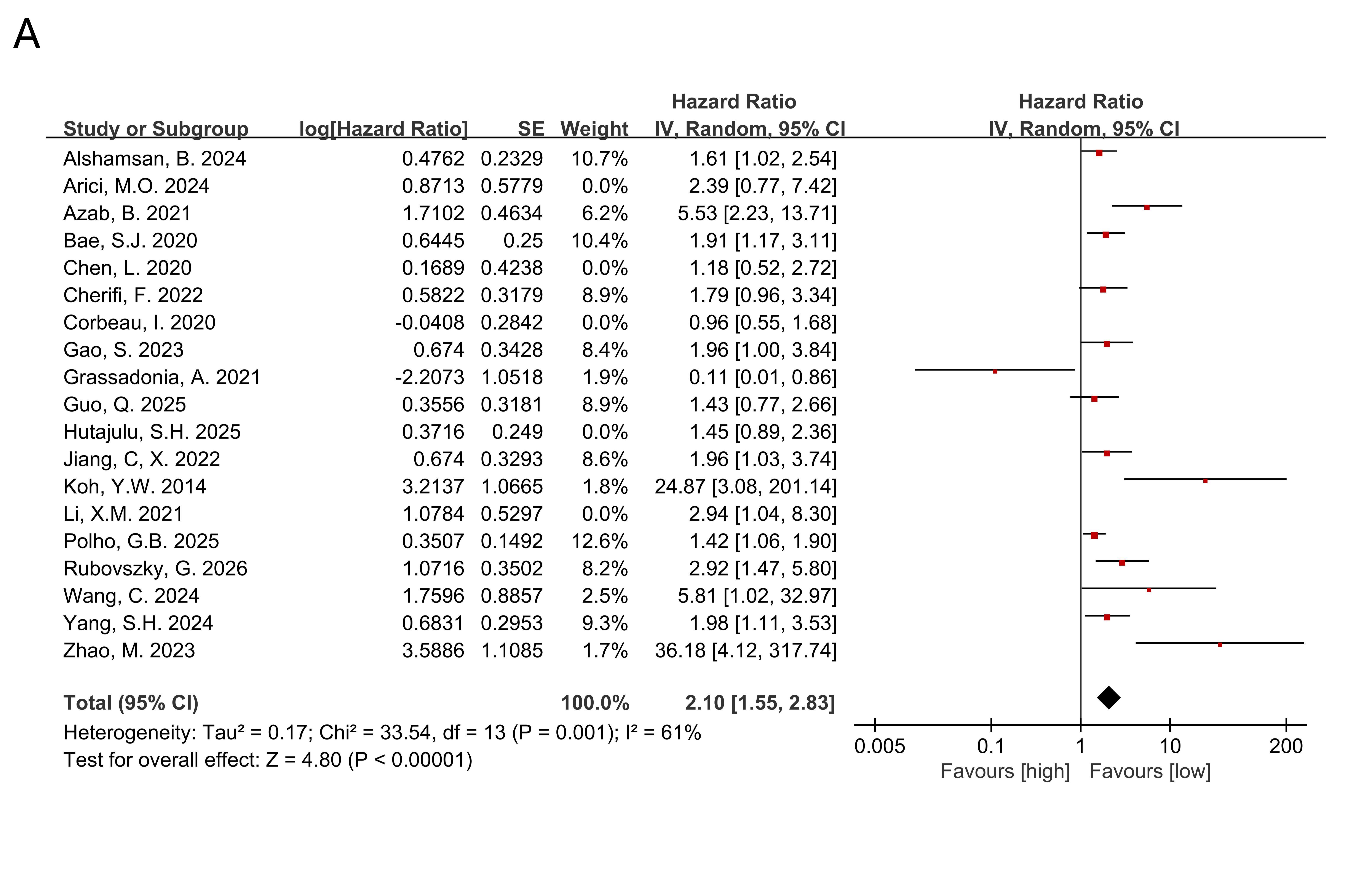

Supplement: Supplementary Figure 1 — Forest plots of multivariable-adjusted associations between elevated pretreatment NLR and clinical outcomes after NACT. Panels A–D show OS, DFS, RFS, and pCR, respectively. HRs were used for survival outcomes, whereas ORs were used for pCR. [file Image1.jpeg]

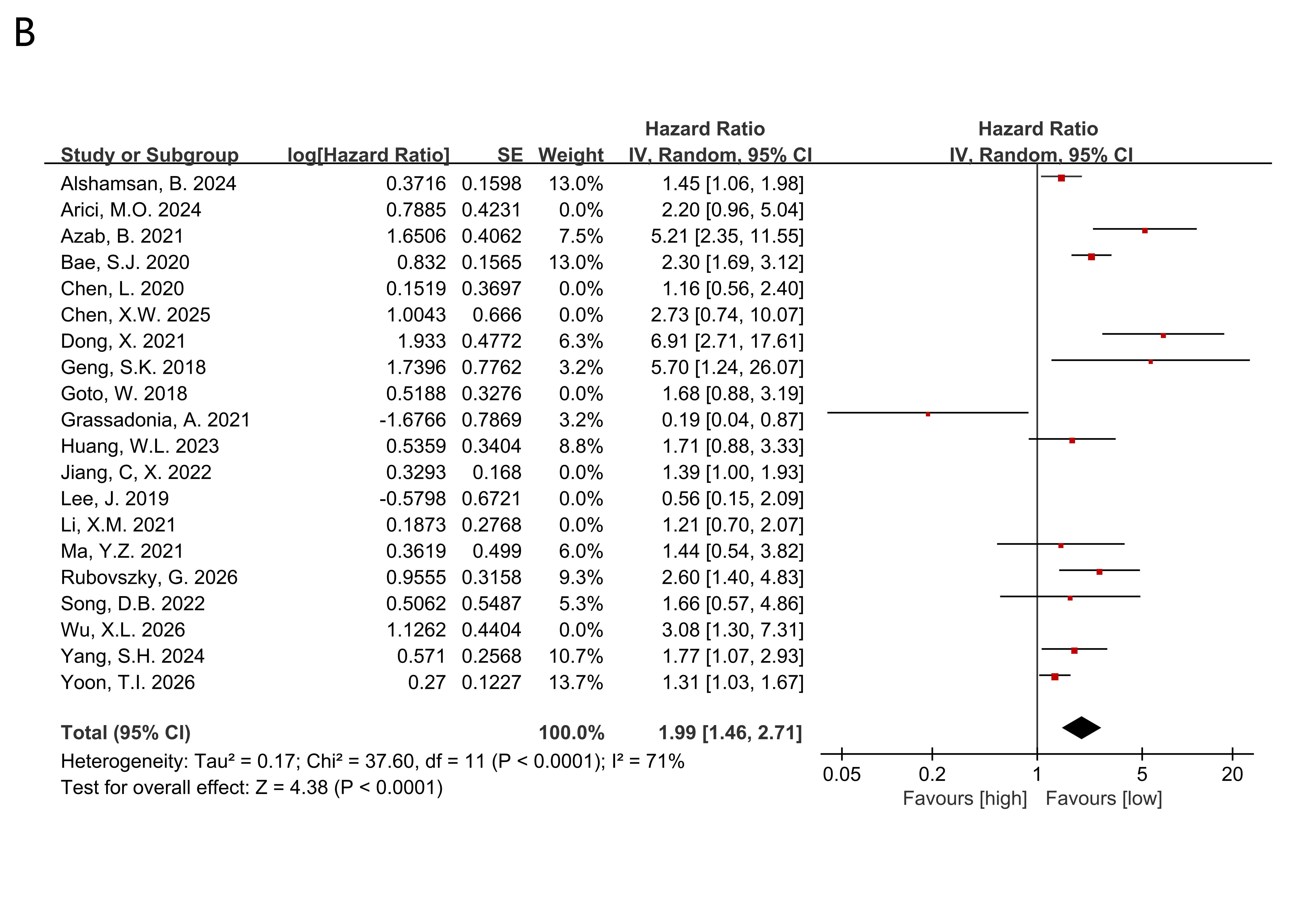

Supplement: Supplementary Figure 2 — Trim-and-fill analyses assessing the potential impact of publication bias. Panel A shows the analysis for OS, and panel B shows the analysis for pCR. [file Image2.jpeg]

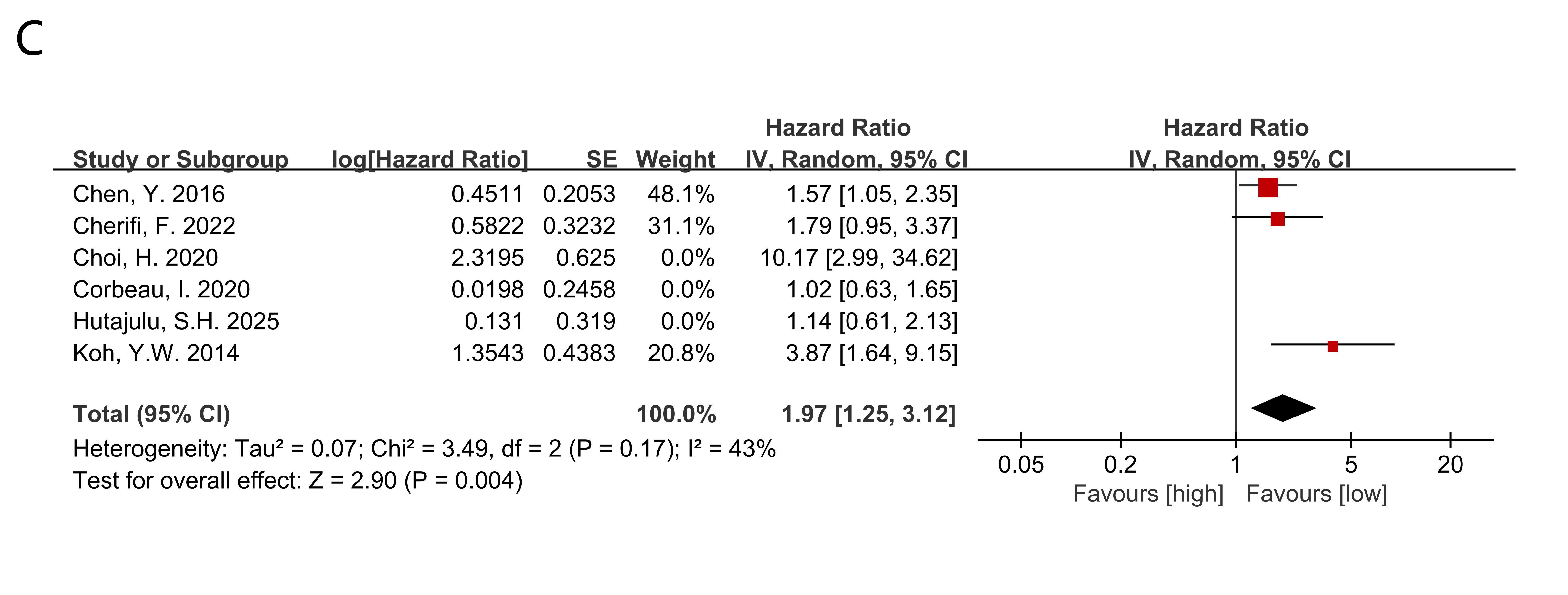

Supplement: Supplementary Figure 3 — Leave-one-out sensitivity analyses of the associations between elevated pretreatment NLR and clinical outcomes after NACT. Panels A–D show OS, DFS, RFS, and pCR, respectively. [file Image3.jpeg]

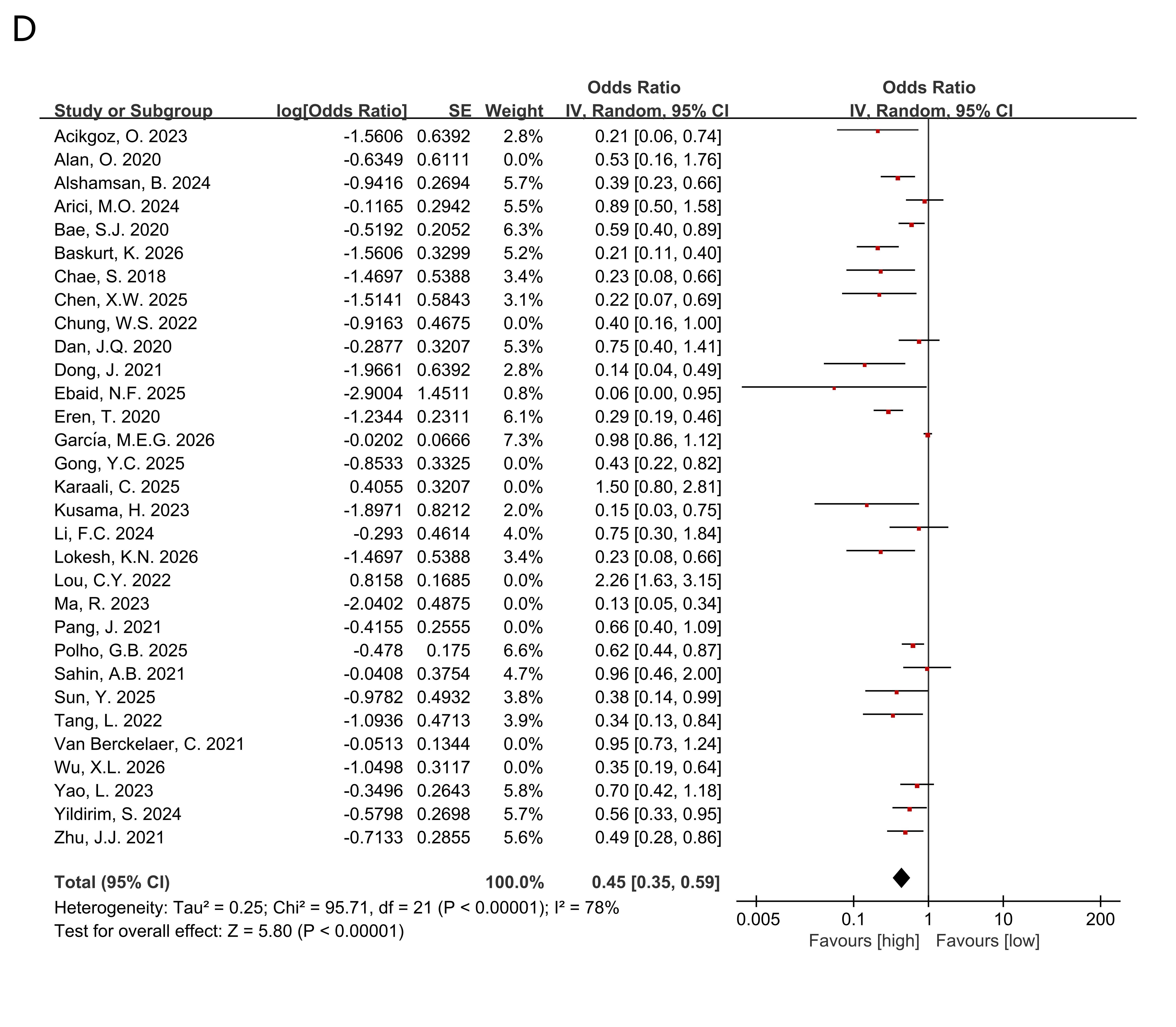

Supplement: Supplementary Figure 4 — Funnel plots for publication bias in the analyses of elevated pretreatment NLR and clinical outcomes after NACT. Panels A–D show OS, DFS, RFS, and pCR, respectively. [file Image4.jpeg]

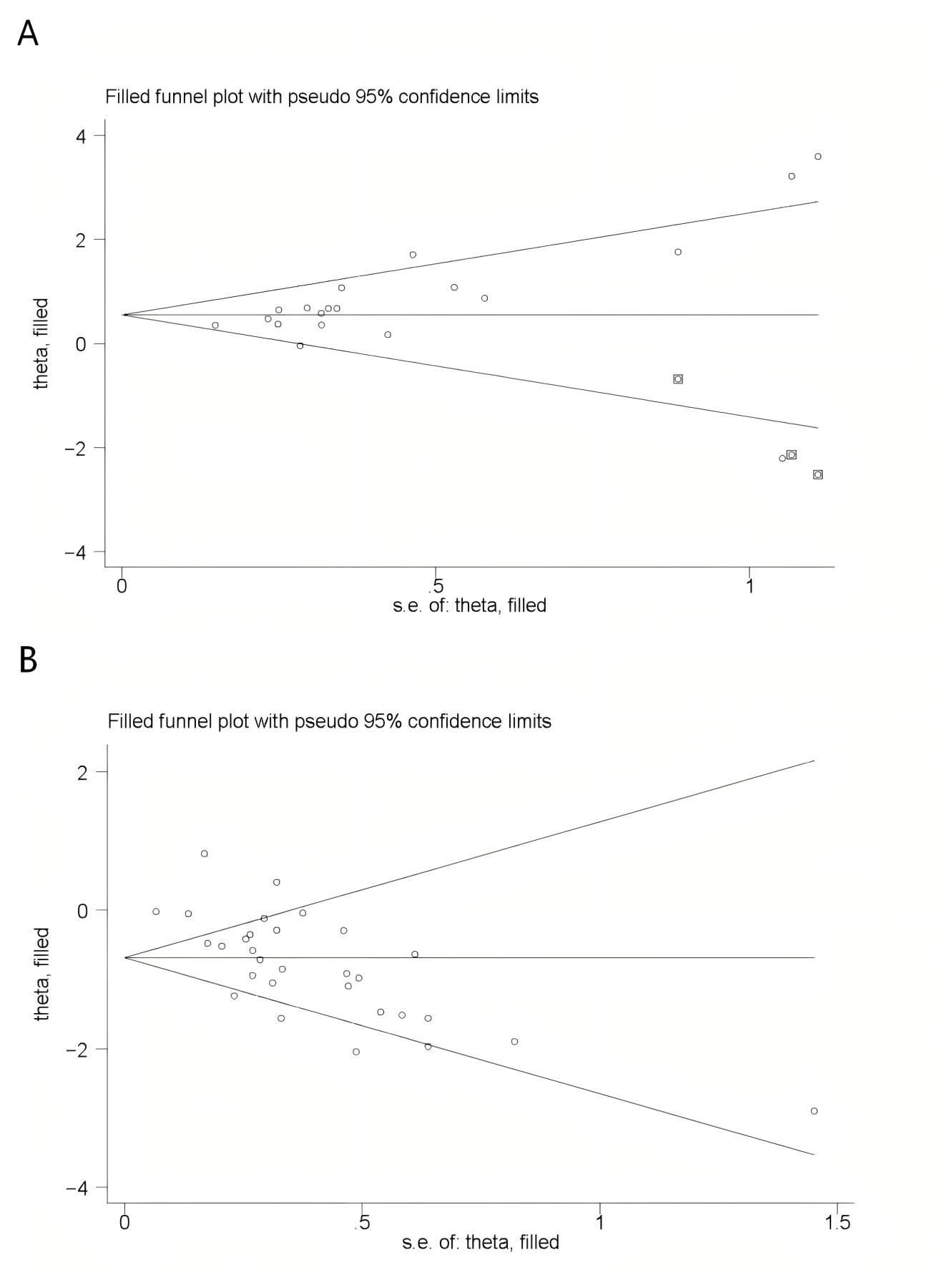

Supplement: Supplementary file 12 [file Image5.jpeg]

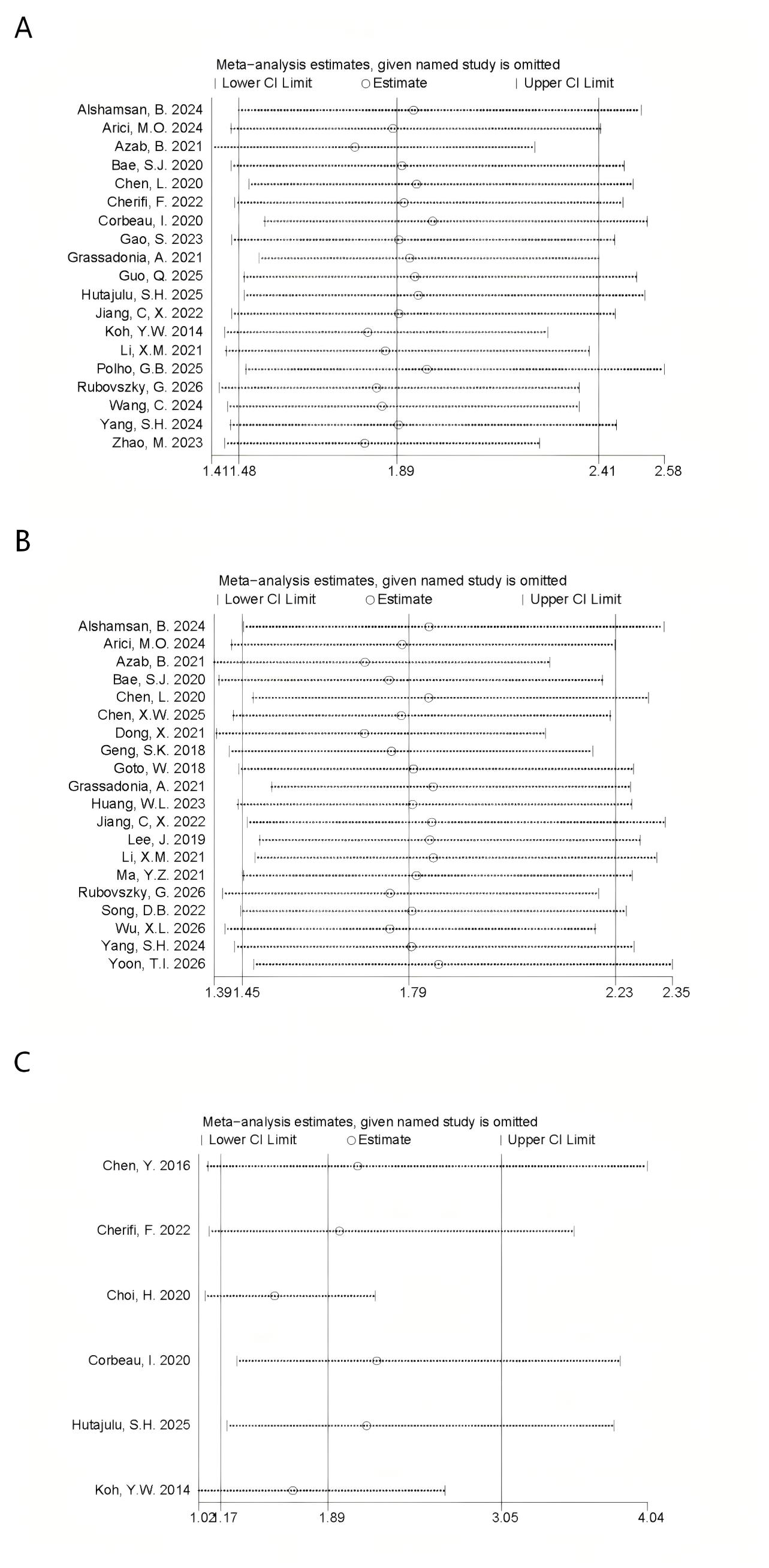

Supplement: Supplementary file 13 [file Image6.jpeg]

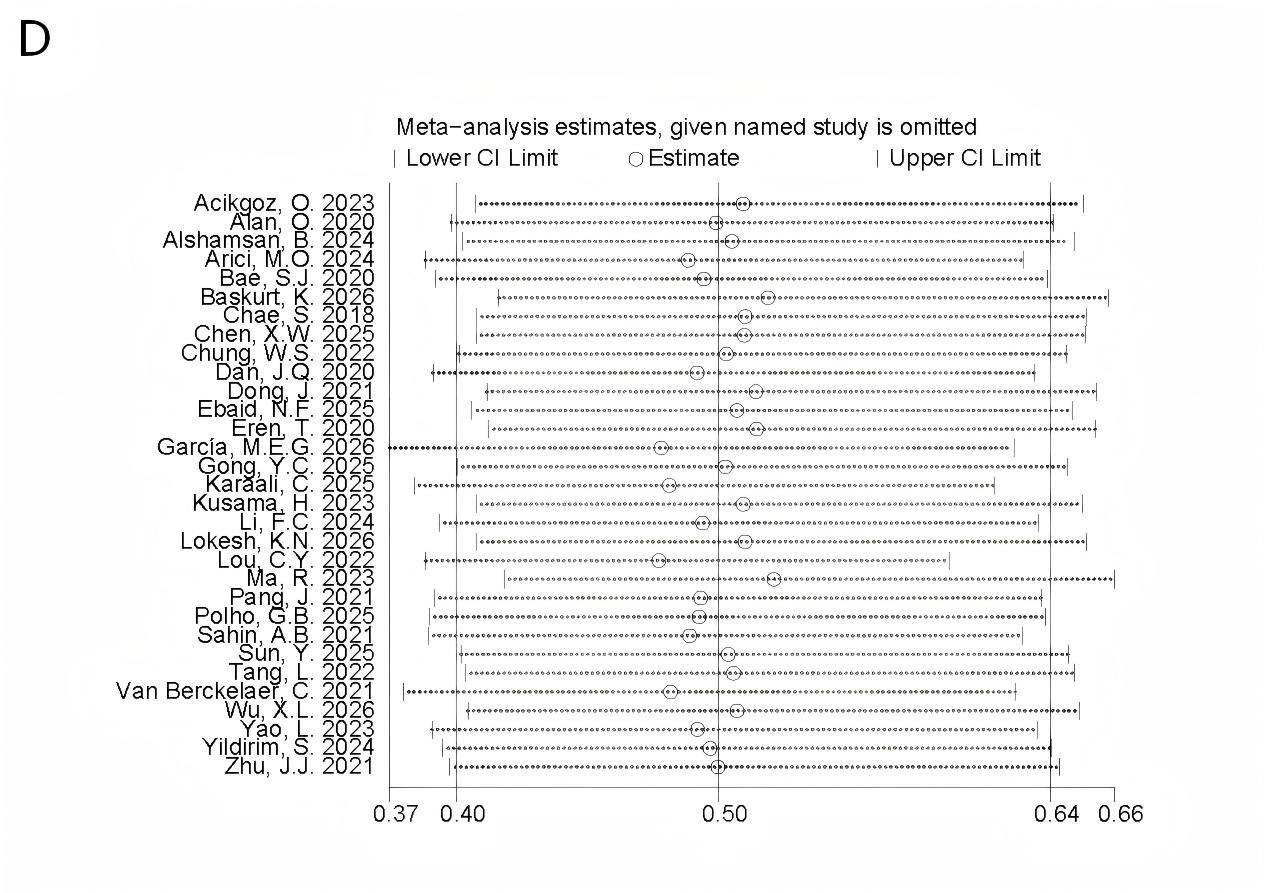

Supplement: Supplementary file 14 [file Image7.jpeg]
