# Supplementary material for: Prognostic value of pretreatment neutrophil-to-lymphocyte ratio in breast cancer patients receiving neoadjuvant chemotherapy: a systematic review and meta-analysis
Source: Front Oncol. 2026 May 29;16:1849765. doi: 10.3389/fonc.2026.1849765 (PMC13260012; doi:10.3389/fonc.2026.1849765)
Supplement: Supplementary Table 1 — Full PubMed search strategy. [file Table1.docx]

| Supplementary Table S1. Detailed search strategy in four databases. | |
| --- | --- |
| Database | Search strategy |
| Pubmed | ((((("Neutrophils"[Mesh]) OR ((((((((Neutrophil) OR (Neutrophils)) OR (Polymorphonuclear Neutrophils)) OR (Polymorphonuclear Neutrophil)) OR (Polymorphonuclear Leukocyte)) OR (Polymorphonuclear Leukocytes)) OR (Neutrophil Band Cells)) OR (Neutrophil Band Cell))) AND (("Lymphocytes"[Mesh]) OR (((Lymphocyte) OR (Lymphoid Cells)) OR (Lymphoid Cell)))) AND (ratio)) AND (("Breast Neoplasms"[Mesh]) OR ((((((((((((((((((((Breast Neoplasm) OR (Breast Tumors)) OR (Breast Tumor)) OR (Breast Cancer)) OR (Cancer of Breast)) OR (Cancer of the Breast)) OR (Malignant Neoplasm of Breast)) OR (Breast Malignant Neoplasm)) OR (Breast Malignant Neoplasms)) OR (Malignant Tumor of Breast)) OR (Breast Malignant Tumor)) OR (Breast Malignant Tumors)) OR (Mammary Cancer)) OR (Mammary Cancers)) OR (Human Mammary Neoplasm)) OR (Human Mammary Neoplasms)) OR (Breast Carcinoma)) OR (Breast Carcinomas)) OR (Human Mammary Carcinomas)) OR (Human Mammary Carcinoma)))) AND (("Neoadjuvant Therapy"[Mesh]) OR (((((((((((((((((((((Neoadjuvant) OR (Neoadjuvant Therapy)) OR (Neoadjuvant Therapies)) OR (Neoadjuvant Treatment)) OR (Neoadjuvant Treatments)) OR (Neoadjuvant Chemotherapy)) OR (Neoadjuvant Chemotherapies)) OR (Neoadjuvant Chemotherapy Treatment)) OR (Neoadjuvant Chemotherapy Treatments)) OR (Neoadjuvant Chemoradiotherapy)) OR (Neoadjuvant Chemoradiotherapies)) OR (Neoadjuvant Chemoradiation)) OR (Neoadjuvant Chemoradiations)) OR (Neoadjuvant Chemoradiation Therapy)) OR (Neoadjuvant Chemoradiation Therapies)) OR (Neoadjuvant Chemoradiation Treatment)) OR (Neoadjuvant Chemoradiation Treatments)) OR (Neoadjuvant Systemic Therapy)) OR (Neoadjuvant Systemic Therapies)) OR (Neoadjuvant Systemic Treatment)) OR (Neoadjuvant Systemic Treatments))) |
| Embase | ((Neutrophils or (Neutrophil or Neutrophils or Polymorphonuclear Neutrophils or Polymorphonuclear Neutrophil or Polymorphonuclear Leukocyte or Polymorphonuclear Leukocytes or Neutrophil Band Cells or Neutrophil Band Cell)) and (Lymphocytes or (Lymphocyte or Lymphoid Cells or Lymphoid Cell)) and Ratio and (Breast Neoplasms or (Breast Neoplasm or Breast Tumors or Breast Tumor or Breast Cancer or Cancer of Breast or Cancer of the Breast or Malignant Neoplasm of Breast or Breast Malignant Neoplasm or Breast Malignant Neoplasms or Malignant Tumor of Breast or Breast Malignant Tumor or Breast Malignant Tumors or Mammary Cancer or Mammary Cancers or Human Mammary Neoplasm or Human Mammary Neoplasms or Breast Carcinoma or Breast Carcinomas or Human Mammary Carcinomas or Human Mammary Carcinoma)) and (Neoadjuvant Therapy or (Neoadjuvant or Neoadjuvant Therapy or Neoadjuvant Therapies or Neoadjuvant Treatment or Neoadjuvant Treatments or Neoadjuvant Chemotherapy or Neoadjuvant Chemotherapies or Neoadjuvant Chemotherapy Treatment or Neoadjuvant Chemotherapy Treatments or Neoadjuvant Chemoradiotherapy or Neoadjuvant Chemoradiotherapies or Neoadjuvant Chemoradiation or Neoadjuvant Chemoradiations or Neoadjuvant Chemoradiation Therapy or Neoadjuvant Chemoradiation Therapies or Neoadjuvant Chemoradiation Treatment or Neoadjuvant Chemoradiation Treatments or Neoadjuvant Systemic Therapy or Neoadjuvant Systemic Therapies or Neoadjuvant Systemic Treatment or Neoadjuvant Systemic Treatments))).af. |
| Web of Science | (((((Neutrophils) OR ((((((((Neutrophil) OR (Neutrophils)) OR (Polymorphonuclear Neutrophils)) OR (Polymorphonuclear Neutrophil)) OR (Polymorphonuclear Leukocyte)) OR (Polymorphonuclear Leukocytes)) OR (Neutrophil Band Cells)) OR (Neutrophil Band Cell))) AND ((Lymphocytes) OR (((Lymphocyte) OR (Lymphoid Cells)) OR (Lymphoid Cell)))) AND (ratio)) AND ((Breast Neoplasms) OR ((((((((((((((((((((Breast Neoplasm) OR (Breast Tumors)) OR (Breast Tumor)) OR (Breast Cancer)) OR (Cancer of Breast)) OR (Cancer of the Breast)) OR (Malignant Neoplasm of Breast)) OR (Breast Malignant Neoplasm)) OR (Breast Malignant Neoplasms)) OR (Malignant Tumor of Breast)) OR (Breast Malignant Tumor)) OR (Breast Malignant Tumors)) OR (Mammary Cancer)) OR (Mammary Cancers)) OR (Human Mammary Neoplasm)) OR (Human Mammary Neoplasms)) OR (Breast Carcinoma)) OR (Breast Carcinomas)) OR (Human Mammary Carcinomas)) OR (Human Mammary Carcinoma)))) AND ((Neoadjuvant Therapy) OR (((((((((((((((((((((Neoadjuvant) OR (Neoadjuvant Therapy)) OR (Neoadjuvant Therapies)) OR (Neoadjuvant Treatment)) OR (Neoadjuvant Treatments)) OR (Neoadjuvant Chemotherapy)) OR (Neoadjuvant Chemotherapies)) OR (Neoadjuvant Chemotherapy Treatment)) OR (Neoadjuvant Chemotherapy Treatments)) OR (Neoadjuvant Chemoradiotherapy)) OR (Neoadjuvant chemoradiotherapic)) OR (Neoadjuvant Chemoradiation)) OR (Neoadjuvant chemoradiation)) OR (Neoadjuvant Chemoradiation Therapy)) OR (Neoadjuvant Chemoradiation Therapies)) OR (Neoadjuvant Chemoradiation Treatment)) OR (Neoadjuvant Chemoradiation Treatments)) OR (Neoadjuvant Systemic Therapy)) OR (Neoadjuvant Systemic Therapies)) OR (Neoadjuvant Systemic Treatment)) OR (Neoadjuvant Systemic Treatments))) (Topic) |
| Chochrane | ((Neutrophils or (Neutrophil or Neutrophils or Polymorphonuclear Neutrophils or Polymorphonuclear Neutrophil or Polymorphonuclear Leukocyte or Polymorphonuclear Leukocytes or Neutrophil Band Cells or Neutrophil Band Cell)) and (Lymphocytes or (Lymphocyte or Lymphoid Cells or Lymphoid Cell)) and ratio and (Breast Neoplasms or (Breast Neoplasm or Breast Tumors or Breast Tumor or Breast Cancer or Cancer of Breast or Cancer of the Breast or Malignant Neoplasm of Breast or Breast Malignant Neoplasm or Breast Malignant Neoplasms or Malignant Tumor of Breast or Breast Malignant Tumor or Breast Malignant Tumors or Mammary Cancer or Mammary Cancers or Human Mammary Neoplasm or Human Mammary Neoplasms or Breast Carcinoma or Breast Carcinomas or Human Mammary Carcinomas or Human Mammary Carcinoma)) and (Neoadjuvant Therapy or (Neoadjuvant or Neoadjuvant Therapy or Neoadjuvant Therapies or Neoadjuvant Treatment or Neoadjuvant Treatments or Neoadjuvant Chemotherapy or Neoadjuvant Chemotherapies or Neoadjuvant Chemotherapy Treatment or Neoadjuvant Chemotherapy Treatments or Neoadjuvant Chemoradiotherapy or Neoadjuvant Chemoradiotherapies or Neoadjuvant Chemoradiation or Neoadjuvant Chemoradiations or Neoadjuvant Chemoradiation Therapy or Neoadjuvant Chemoradiation Therapies or Neoadjuvant Chemoradiation Treatment or Neoadjuvant Chemoradiation Treatments or Neoadjuvant Systemic Therapy or Neoadjuvant Systemic Therapies or Neoadjuvant Systemic Treatment or Neoadjuvant Systemic Treatments))).af. |

Note: The search strategies were adapted for each database using controlled vocabulary and free-text terms related to neutrophils, lymphocytes, breast cancer, and neoadjuvant therapy.
