# Supplementary material for: Prognostic value of pretreatment neutrophil-to-lymphocyte ratio in breast cancer patients receiving neoadjuvant chemotherapy: a systematic review and meta-analysis
Source: Front Oncol. 2026 May 29;16:1849765. doi: 10.3389/fonc.2026.1849765 (PMC13260012; doi:10.3389/fonc.2026.1849765)
Supplement: Supplementary Table 2 — Quality assessment of the included studies using the Newcastle–Ottawa Scale. [file Table2.docx]

| Supplementary Table S2. Quality evaluation of the eligible studies with Newcastle–Ottawa scale. | | | | | | | | | |
| --- | --- | --- | --- | --- | --- | --- | --- | --- | --- |
| Study | Selection | | | | Comparability | | Outcome | | |
|  | Representative-ness | Selection of  non-exposed | Ascertainment  of exposure | Outcome not present at start | Comparability on most important factors | Comparability on other risk factors | Assessment of outcome | Long enough follow-up (median≥1 year) | Adequacy  (completeness) of follow-up |
| Acikgoz, O. 2023 | - | * | * | * | - | - | * | * | * |
| Alan, O. 2020 | - | * | * | * | - | - | * | * | * |
| Alshamsan, B. 2024 | - | * | * | * | - | * | * | * | * |
| Arici, M.O. 2024 | * | * | * | * | - | - | * | * | * |
| Azab, B. 2021 | - | - | * | * | - | - | * | * | * |
| Bae, S.J. 2020 | - | * | * | * | - | * | * | * | * |
| Baskurt, K. 2026 | * | * | * | * | - | - | * | * | * |
| Chae, S. 2018 | - | * | * | * | - | - | * | * | * |
| Chen, L. 2020 | - | * | * | * | - | * | * | * | * |
| Chen, X.W. 2025 | - | * | * | * | - | * | * | * | * |
| Chen, Y. 2016 | * | * | * | * | - | - | * | * | * |
| Cherifi, F. 2022 | - | - | * | * | - | - | * | * | * |
| Choi, H. 2020 | - | * | * | * | - | - | * | * | * |
| Chung, W.S. 2022 | * | * | * | * | - | - | * | - | * |
| Corbeau, I. 2020 | * | - | * | * | - | - | * | * | * |
| Dan, J.Q. 2020 | * | * | * | * | - | - | * | * | * |
| Dong, J. 2021 | * | * | * | * | - | - | * | - | * |
| Dong, X. 2021 | - | * | * | * | * | * | * | * | * |
| Ebaid, N.F. 2025 | * | * | * | * | - | - | * | - | * |
| Eren, T. 2020 | - | * | * | * | - | * | * | * | * |
| Gao, S. 2023 | * | * | * | * | * | * | * | * | * |
| García, M.E.G. 2026 | * | - | * | * | - | - | * | * | * |
| Geng, S.K. 2018 | * | * | * | * | - | - | * | * | * |
| Gong, Y.C. 2025 | * | * | * | * | * | - | * | - | * |
| Goto, W. 2018 | * | * | * | * | - | - | * | * | * |
| Grassadonia, A. 2021 | * | - | * | * | - | * | * | * | * |
| Guo, Q. 2025 | - | * | * | * | - | - | * | * | * |
| Huang, W.L. 2023 | * | * | * | * | - | - | * | * | * |
| Hutajulu, S.H. 2025 | * | * | * | * | - | * | * | * | * |
| Jiang, C, X. 2022 | * | * | * | * | * | * | * | - | * |
| Karaali, C. 2025 | * | * | * | * | - | - | * | - | * |
| Koh, Y.W. 2014 | * | * | * | * | * | * | * | * | * |
| Kusama, H. 2023 | - | * | * | * | - | - | * | * | * |
| Lee, J. 2019 | - | * | * | * | - | * | * | * | * |
| Li, F.C. 2024 | - | * | * | * | - | - | * | * | * |
| Li, X.M. 2021 | * | * | * | * | - | * | * | * | * |
| Lokesh, K.N. 2026 | - | * | * | * | - | - | * | * | * |
| Lou, C.Y. 2022 | - | * | * | * | - | - | * | * | * |
| Ma, R. 2023 | * | * | * | * | - | - | * | * | * |
| Ma, Y.Z. 2021 | * | * | * | * | - | * | * | * | * |
| Pang, J. 2021 | - | * | * | * | * | * | * | * | * |
| Polho, G.B. 2025 | - | - | * | * | - | - | * | * | * |
| Rubovszky, G. 2026 | - | * | * | * | - | * | * | * | * |
| Sahin, A.B. 2021 | * | * | * | * | - | - | * | * | * |
| Song, D.B. 2022 | * | * | * | * | - | * | * | * | * |
| Sun, Y. 2025 | * | * | * | * | - | * | * | * | * |
| Tang, L. 2022 | * | * | * | * | - | * | * | * | * |
| Van Berckelaer, C. 2021 | - | - | * | * | - | * | * | * | * |
| Wang, C. 2024 | - | * | * | * | * | * | * | * | * |
| Wu, X.L. 2026 | - | - | * | * | - | - | * | * | * |
| Yang, S.H. 2024 | * | * | * | * | - | * | * | * | * |
| Yao, L. 2023 | * | - | * | * | - | - | * | * | * |
| Yildirim, S. 2024 | * | * | * | * | - | - | * | * | * |
| Yoon, T.I. 2026 | * | * | * | * | - | - | * | * | * |
| Zhao, M. 2023 | - | * | * | * | - | - | * | * | * |
| Zhu, J.J. 2021 | * | * | * | * | - | * | * | * | * |
| *indicates criterion met; - indicates significant of criterion not met. | | | | | | | | | |
