# Supplementary material for: Prognostic value of pretreatment neutrophil-to-lymphocyte ratio in breast cancer patients receiving neoadjuvant chemotherapy: a systematic review and meta-analysis
Source: Front Oncol. 2026 May 29;16:1849765. doi: 10.3389/fonc.2026.1849765 (PMC13260012; doi:10.3389/fonc.2026.1849765)
Supplement: Supplementary Table 3 — Cross-checking table for potential cohort overlap among included studies. [file Table3.docx]

| Author | study period | region | Center / hospital | Population | Outcomes | Overlap assessment |
| --- | --- | --- | --- | --- | --- | --- |
| Acikgoz, O. 2023 | 2014-2019 | Turkey | Istanbul Medipol University School of Medicine | Locally advanced breast cancer | pCR | No apparent overlap |
| Alan, O. 2020 | 2015-2017 | Turkey | Marmara University School of Medicine | Locally advanced and metastatic breast cancer | pCR | No apparent overlap |
| Arici, M.O. 2024 | 2015-2023 | Turkey | Antalya Training and Research Hospital | Early or locally advanced breast cancer | OS/DFS/pCR | No apparent overlap |
| Baskurt, K. 2026 | 2022-2024 | Turkey | Etlik City Hospital | HR +/HER2 − BC | pCR | No apparent overlap |
| Chen, L. 2020 | 1999-2014 | China | Cancer Hospital Chinese Academy of Medical Sciences | Advanced BC | OS/DFS | No apparent overlap |
| Chen, X.W. 2025 | 2019-2023 | China | Sun Yat-sen University Cancer Center | TNBC | DFS/pCR | Different study periods：Chen, X.W. 2025 2019-2023  vs Huang, W.L. 2023 2014-2018 |
| Huang, W.L. 2023 | 2014-2018 | China | Sun Yat-sen University Cancer Center | BC | DFS |  |
| Chen, Y. 2016 | 2001-2010 | China | Sun Yat-sen Memorial Hospital | Primary breast cancer | RFS | No apparent overlap |
| Chung, W.S. 2022 | 2012-2019 | China | Linkuo Chang Gung Memorial Hospital | Primary non-metastatic TNBC | pCR | No apparent overlap |
| Dan, J.Q. 2020 | 2012-2017 | China | Chengdu Fifth People's Hospital | Primary breast cancer | pCR | No apparent overlap |
| Dong, J. 2021 | 2015-2020 | China | the First Affiliated Hospital of USTC | Primary breast cancer | pCR | No apparent overlap |
| Dong, X. 2021 | 2010-2014 | China | Xiangya Hospital, Central South University | TNBC | DFS | Different outcomes：Dong, X. 2021 DFS  vs Pang, J. 2021 pCR |
| Pang, J. 2021 | 2010-2018 | China | Xiangya Hospital, Central South University | Locally advanced TNBC | pCR |  |
| Eren, T. 2020 | 2009-2018 | Turkey | Medical Oncology Clinic of Ankara Numune Training and Research Hospital | Locally advanced breast cancer | pCR | No apparent overlap |
| Gao, S. 2023 | 2009-2018 | China | Shandong First Medical University and Shandong Academy of Medical Sciences | BC | OS | No apparent overlap |
| Geng, S.K. 2018 | 2002-2014 | China | Zhongshan Hospital, Fudan University | BC | DFS | No apparent overlap |
| Gong, Y.C. 2025 | 1999-2018 | China | The First Affiliated Hospital of Nanjing Medical University | BC | pCR | No apparent overlap |
| Guo, Q. 2025 | 2000-2018 | China | Inner Mongolia People's Hospital | TNBC | OS | No apparent overlap |
| Jiang, C, X. 2022 | 2012-2016 | China | Tumor Hospital Affiliated to Harbin Medical University | BC | OS/DFS | Different outcomes：Jiang, C, X. 2022 OS/DFS  vs Li, F.C. 2024 pCR |
| Li, F.C. 2024 | 2011-2023 | China | Tumor Hospital Affiliated to Harbin Medical University | Young patients with breast cancer | pCR |  |
| Karaali, C. 2025 | 2010-2021 | Turkey | University of Health Sciences, Izmir Faculty of Medicine | BC | pCR | No apparent overlap |
| Li, X.M. 2021 | 2008-2018 | China | West China Hospital, Sichuan University | Primary breast cancer | OS/DFS | No apparent overlap |
| Lou, C.Y. 2022 | 2015-2021 | China | People’s Hospital of Zhuji | TNBC | pCR | No apparent overlap |
| Ma, R. 2023 | 2019-2022 | China | the First Affiliated Hospital of Xi’an Jiaotong University | BC | pCR | No apparent overlap |
| Ma, Y.Z. 2021 | 2017-2018 | China | Henan Cancer Hospital | BC | DFS | Different outcomes：Ma, Y.Z. 2021 DFS  vs Zhu, J.J. 2021 pCR |
| Zhu, J.J. 2021 | 2014-2019 | China | Henan Cancer Hospital | Invasive breast cancer | pCR |  |
| Sahin, A.B. 2021 | 2008-2019 | Turkey | Uludag University Medical Center | BC | pCR | No apparent overlap |
| Song, D.B. 2022 | 2016-2018 | China | Shangqiu First People’s Hospital | BC | DFS | No apparent overlap |
| Sun, Y. 2025 | 2010-2020 | China | Nanjing Drum Tower Hospital | Invasive breast cancer | pCR | No apparent overlap |
| Tang, L. 2022 | 2012-2019 | China | the First Affiliated Hospital of Chongqing Medical University | HR +/HER2 − BC | pCR | No apparent overlap |
| Wang, C. 2024 | 2014-2019 | China | Southwest Hospital, Army Medical University | TNBC | OS | No apparent overlap |
| Yildirim, S. 2024 | 2010-2022 | Turkey | Lütfi Kirdar City Hospital, Health Science University | BC | pCR | No apparent overlap |
| Zhao, M. 2023 | 2017-2018 | China | The Fourth Hospital of Hebei Medical University | TNBC | OS | No apparent overlap |

Note: This table was used to assess potential cohort overlap among studies from similar countries, centers, study periods, or patient populations. “No apparent overlap” indicates that no clear evidence of duplicate patient inclusion was identified.
