# Supplementary material for: Prognostic value of pretreatment neutrophil-to-lymphocyte ratio in breast cancer patients receiving neoadjuvant chemotherapy: a systematic review and meta-analysis
Source: Front Oncol. 2026 May 29;16:1849765. doi: 10.3389/fonc.2026.1849765 (PMC13260012; doi:10.3389/fonc.2026.1849765)
Supplement: Supplementary Table 4 — Univariable and multivariable meta-regression analyses for OS. [file Table4.docx]

| Variables | Coefficient | P value | 95% CI | R² | τ² |
| --- | --- | --- | --- | --- | --- |
| **Sample size** |  |  |  |  |  |
| Per 1-patient increase | -0.00030 | 0.351 | [-0.00094,0.00034] | -60.20% | 0.052 |
| **Follow-up** |  |  |  |  |  |
| Per 1-month increase | -0.013 | 0.082 | [-0.028,0.002] | -343.20% | 0.144 |
| **Mean/Median Age** |  |  |  |  |  |
| Per 1-year increase | 0.019 | 0.626 | [-0.058,0.097] | -20.10% | 0.039 |
| **NLR cut-off** |  |  |  |  |  |
| Per 1-unit increase | 0.207 | 0.126 | [-0.058,0.473] | 97.96% | 0.00066 |
| **Region** |  |  |  |  |  |
| Non-Asia vs Asia | 0.061 | 0.819 | [-0.459,0.580] | -17.30% | 0.038 |
| Multicenter vs Asia | -0.214 | 0.296 | [-0.614,0.187] |  |  |
| **Population** |  |  |  |  |  |
| Triple-negative breast cancer vs Unselected breast cancer | 0.201 | 0.476 | [-0.352,0.754] | -161.70% | 0.085 |
| Other specific breast cancer vs Unselected breast cancer | -0.040 | 0.906 | [-0.694,0.615] |  |  |
| Stage-specific breast cancer vs Unselected breast cancer | -0.335 | 0.267 | [-0.925,0.256] |  |  |
| **Tumor stage** |  |  |  |  |  |
| Mixed vs Non-metastatic | 1.498 | 0.001 | [0.647,2.349] | 100% | 0 |
| **Adjustment** |  |  |  |  |  |
| Multivariate vs Univariate | 0.315 | 0.124 | [-0.086,0.716] | 19.14% | 0.026 |

Note: This table presents univariable meta-regression results for OS. The coefficient represents the change in the log HR associated with each covariate. For categorical variables, the first category listed in the comparison was used as the reference group. R² indicates the proportion of between-study heterogeneity explained by the covariate, and τ² represents the residual between-study variance. Results should be interpreted cautiously because meta-regression was based on study-level data.

| Variables | Coefficient | P value | 95% CI | R² | τ² |
| --- | --- | --- | --- | --- | --- |
| **NLR cut-off** |  |  |  |  |  |
| Per 1-unit increase | 0.200 | 0.148 | [-0.058,0.473] | 100.00% | 0.00 |
| **Tumor stage** |  |  |  |  |  |
| Mixed vs Non-metastatic | 1.487 | 0.001 | [0.647,2.349] |  |  |
| **Adjustment** |  |  |  |  |  |
| Multivariate vs Univariate | 0.168 | 0.359 | [-0.190,0.526] |  |  |

Note: This table presents multivariable meta-regression results for OS. The coefficient represents the change in the log HR after simultaneous adjustment for the listed covariates. R² indicates the proportion of between-study heterogeneity explained by the model, and τ² represents the residual between-study variance.
