# Supplementary material for: Prognostic value of pretreatment neutrophil-to-lymphocyte ratio in breast cancer patients receiving neoadjuvant chemotherapy: a systematic review and meta-analysis
Source: Front Oncol. 2026 May 29;16:1849765. doi: 10.3389/fonc.2026.1849765 (PMC13260012; doi:10.3389/fonc.2026.1849765)
Supplement: Supplementary Table 7 — Definitions of pathological complete response reported in the included studies. [file Table7.docx]

| Author | Definition of pCR in the original study |
| --- | --- |
|  |  |
| Acikgoz, O. 2023 | tpCR |
| Alan, O. 2020 | Not clearly reported |
| Alshamsan, B. 2024 | tpCR |
| Arici, M.O. 2024 | tpCR |
| Azab, B. 2021 | NA |
| Bae, S.J. 2020 | Not clearly reported |
| Baskurt, K. 2026 | tpCR |
| Chae, S. 2018 | tpCR |
| Chen, L. 2020 | NA |
| Chen, X.W. 2025 | tpCR |
| Chen, Y. 2016 | NA |
| Cherifi, F. 2022 | NA |
| Choi, H. 2020 | NA |
| Chung, W.S. 2022 | tpCR |
| Corbeau, I. 2020 | NA |
| Dan, J.Q. 2020 | tpCR |
| Dong, J. 2021 | tpCR |
| Dong, X. 2021 | NA |
| Ebaid, N.F. 2025 | tpCR |
| Eren, T. 2020 | tpCR |
| Gao, S. 2023 | NA |
| García, M.E.G. 2026 | tpCR |
| Geng, S.K. 2018 | NA |
| Gong, Y.C. 2025 | tpCR |
| Goto, W. 2018 | NA |
| Grassadonia, A. 2021 | NA |
| Guo, Q. 2025 | NA |
| Huang, W.L. 2023 | NA |
| Hutajulu, S.H. 2025 | NA |
| Jiang, C, X. 2022 | NA |
| Karaali, C. 2025 | tpCR |
| Koh, Y.W. 2014 | NA |
| Kusama, H. 2023 | ypT0/is ypN0 or JBCS grade ≥2b |
| Lee, J. 2019 | NA |
| Li, F.C. 2024 | tpCR |
| Li, X.M. 2021 | NA |
| Lokesh, K.N. 2026 | tpCR |
| Lou, C.Y. 2022 | Not clearly reported |
| Ma, R. 2023 | Not clearly reported |
| Ma, Y.Z. 2021 | NA |
| Pang, J. 2021 | Not clearly reported |
| Polho, G.B. 2025 | tpCR |
| Rubovszky, G. 2026 | NA |
| Sahin, A.B. 2021 | tpCR |
| Song, D.B. 2022 | NA |
| Sun, Y. 2025 | tpCR |
| Tang, L. 2022 | tpCR |
| Van Berckelaer, C. 2021 | tpCR |
| Wang, C. 2024 | NA |
| Wu, X.L. 2026 | tpCR |
| Yang, S.H. 2024 | NA |
| Yao, L. 2023 | tpCR |
| Yildirim, S. 2024 | tpCR |
| Yoon, T.I. 2026 | NA |
| Zhao, M. 2023 | NA |
| Zhu, J.J. 2021 | tpCR |

Note: pCR, pathological complete response; tpCR, total pathological complete response; JBCS, Japanese Breast Cancer Society; NA, not applicable because the study did not contribute data to the pCR analysis. “Not clearly reported” indicates that the original study reported pCR but did not provide an explicit pathological definition.
